# Beclin-1 is a Promising Prognostic Biomarker in a Specific Esophageal Squamous Cell Carcinoma Population

**DOI:** 10.3389/pore.2021.594724

**Published:** 2021-04-09

**Authors:** Hailei Du, Fangxiu Luo, Minmin Shi, Jiaming Che, Lianggang Zhu, Hecheng Li, Junbiao Hang

**Affiliations:** ^1^ Department of Thoracic Surgery, Ruijin Hospital, Shanghai Jiao Tong University School of Medicine, Shanghai, China; ^2^ Department of Pathology, Ruijin Hospital North, Shanghai Jiao Tong University School of Medicine, Shanghai, China; ^3^ Institute of Digestive Surgery, Shanghai, China

**Keywords:** esophageal squamous cell carcinoma (ESCC), autophagy, apoptosis, beclin-1, Prognosis

## Abstract

The effects of autophagy and apoptosis in the prognostic assessment and treatment of Esophageal squamous cell carcinoma (ESCC) remain to be elucidated. Here, we conducted a retrospective study on the histopathology of ESCC, investigated the expression of Beclin-1 and Bcl-2 proteins (both autophagy- and apoptosis-related) in esophageal cancer tissue, and analyzed the significance of these proteins for the prognosis of ESCC. In the present study, the expression level of Beclin-1 in ESCC was significantly lower than that in adjacent tissues (*p* < 0.01), whereas the expression level of Bcl-2 showed the opposite pattern (*p* < 0.01). Furthermore, low expression of Beclin-1 was associated with more advanced ESCC stages and lymph node metastasis. However, high expression of Bcl-2 was associated with more advanced ESCC stages, deeper tumor invasion, and lymph node metastasis. Moreover, the relationship between Bcl-2 expression and OS was not significant (*p* > 0.05), whereas Beclin-1 expression was significantly associated with OS (*p* < 0.05). Subgroup analysis showed that Beclin-1 expression was significantly associated with OS in the high-Bcl-2-expression group but not in the low-Bcl-2-expression group. Importantly, Beclin-1 upregulation or downregulation significantly upregulated or downregulated invasion, respectively, in EC9706 cells in combination with high expression but not low expression of Bcl-2. These findings reveal that differences in autophagy and apoptotic states and their activities may promote malignant tumor differentiation, which could lead to a more aggressive esophageal squamous cell phenotype and a worse survival prognosis. Here, Beclin-1 was shown to be a promising prognostic biomarker and therapeutic target for patients with ESCC in the high-Bcl-2-expression population.

## Introduction

Esophageal cancer is one of the most invasive cancers, and consequently ranks as the sixth most common cause of cancer death worldwide [1]. Esophageal squamous cell carcinoma (ESCC), which is the main histological subtype of esophageal cancer with almost 90% of esophageal cancer being ESCC [2], has high morbidity and mortality. Despite improved diagnostic and treatment strategies such as endoscopic resection, targeted therapy, and immunotherapy, the overall survival (OS) of ESCC patients remains poor. For example, the 5-years survival rate for patients with advanced esophageal cancer is <20% [3]. Therefore, to improve the prognosis of patients with ESCC, it is important to understand how to improve early diagnosis, identify new prognostic markers, and find new therapeutic targets.

Autophagy is a conservative pathway that exists in eukaryotic cells [4]. It degrades and removes damaged organelle components in cells to maintain the homeostasis of cells and provides nutritional support for cell survival [5]. Previous studies have found that abnormal autophagy is associated with a variety of human diseases such as Alzheimer’s disease, cardiovascular disease, and several types of cancer including ESCC [6–8]. As the first autophagy-specific gene identified, Beclin-1 plays an important role in the regulation of autophagy. Changes in Beclin-1 expression levels have been described in a variety of cancers [9–11]. However, the association between Beclin-1 and the pathogenesis of tumors is yet to be studied in detail, and correlation results between the two remain controversial.

B-cell lymphoma-2 (Bcl-2) protein is a regulator of both autophagy and apoptosis. There are two sources of Bcl-2: Bcl-2 in the ER (ER-Bcl-2) and mitochondrial Bcl-2 (mito-Bcl-2). Research indicates that these Bcl-2 proteins from different sources perform different roles in the regulation of autophagy and apoptosis [12]. Therefore, investigating how different Bcl-2 expression levels shift from regulating autophagy to apoptosis is important. In addition, the relationship between Beclin-1 and Bcl-2 in ESCC has not yet been fully elucidated.

Therefore, the central purpose of our study was to analyze the relationships between both Beclin-1 and Bcl-2 and the clinicopathological characteristics of patients with ESCC. Furthermore, we investigated the effect of Beclin-1 on the prognosis of ESCC patients with different expression levels of Bcl-2.

## Methods and Materials

### Patients and Tissue Samples

Patients who underwent surgical resection of esophageal cancer at the Shanghai Ruijin hospital from July 2013 to July 2014 were selected for this study. All patients were diagnosed with ESCC after surgery and patients who received neoadjuvant therapy or showed postoperative complications were excluded: 102 patients with ESCC were ultimately included. All tissue specimens were acquired from pathological paraffin sections. The study group included 65 males and 37 females (age: 45–75 years; median age: 63.5 years). Patients were classified into stages I, II, III, and IV according to the eighth edition of the TNM staging of esophageal cancer. The follow-up observation endpoint was July 2019. This study was approved by the Clinical Ethics Committee of Ruijin hospital.

### Immunohistochemistry (IHC) Staining and Evaluation

Formalin-fixed and paraffin-embedded 5-μm ESCC tissues were deparaffinized in xylene, and then sequentially rehydrated in a range of alcohol concentrations before being washed three times with PBS. IHC staining was performed using primary anti-Beclin-1 (1:200 dilution; Santa Cruz, SC-11427) and anti-Bcl-2 (1:100 dilution; Abcam, ab32124) incubated at 4°C overnight. Slices were then incubated for 15 min with a biotin-labeled secondary antibody before being washed with PBS three times. Hematoxylin staining was performed for 2 min to counterstain. Two different pathologists judged the intensity of the immunostaining in a blinded manner. Staining was scored separately using two methods as follows. Scoring standard A: ≤10% positive cells = 1 point; 11%–50% positive cells = 2 points; 51–75% positive cells = 3 points; >75% positive cells = 4 points. Staining intensity score B: 0 = negative; 1 = light yellow; 2 = brownish-yellow; 3 = brownish. The terminal score was determined as A × B. Where this score was ≤3, the subject was classified into the low expression group; where this score was >3, the subject was classified into high expression group.

### Small Interfering RNA (siRNA)

Bcl-2 siRNA (cat. no. 29214, Santa Cruz) and Beclin-1 siRNA (cat. no. 137198, Invitrogen) were transfected using the Lipofectamine RNAiMAX Transfection reagent (cat. no. 13778-075, Invitrogen) according to the recommended protocol. After 24 h, Bcl-2 and Beclin-1 western blotting was performed to detect the respective expression of each protein. Control Bcl-2 siRNA (cat. no. 37007, Santa Cruz) and control Beclin-1 siRNA (cat. no. 12935400, Invitrogen) were used as controls, respectively.

### Construction of Overexpression Vectors

Recombinant pcDNA3.1-Bcl-2 plasmid and pcDNA3.1-Beclin-1 plasmid (provided by Dr. Qin Ye and Ruyuan Zhang, respectively, Shanghai Institute of Digestive Surgery, Shanghai Jiao Tong University School of Medicine) were transfected into human esophageal carcinoma EC9706 cells by lipofectamine 2000. Bcl-2 and Beclin-1 western blotting was then performed as previously described protocols [13].

### Isolation of Proteins and Western Blot Analysis

The cells were lyzed in lysis buffer and then high-speed centrifuged at 12,000 rpm, for 10 min. A BCA assay was used to quantify protein concentrations. The supernatant was separated on 12.5% SDS-PAGE gel and transferred to a polyvinylidene membrane. After transfer, the membrane was blocked with 5% non-fat milk and incubated with specific primary antibodies as follows. Incubation with Beclin-1 (cat. no. 48341, Santa Cruz) and Bcl-2 (cat. no. 509, Santa Cruz) was conducted overnight at 4°C. After incubation with a secondary antibody, the specific proteins were detected by enhanced chemical luminescence.

### Transwell Migration Assay

An 8-µm inserted Transwell chamber (Corning Incorporated, NY, United States) was used to examine cell migration with or without matrigel. First, 6 × 104 cells in 200 µL of DMEM without serum were added to the upper chamber, while 600 µL of DMEM medium with 10% FBS was placed in the bottom wells. After 24 h incubation at 37°C, cells that had not moved to the lower chamber were removed. Subsequently, Crystal violet was used to stain the cells for 15 min before they were washed with PBS. Finally, five 200 optical fields were taken to count the number of invading cells. The average number of cells was calculated: data represent the mean ± standard deviation.

### Clinical Outcome Assessment

OS was defined as survival from the date of surgery to the date of death due to cancer. The OS of patients with ESCC was acquired in 102 cases: the mean OS was 40.60 ± 2.11 months (median: 31 months; range: 18–72 months).

### Statistical Analysis

Data were statistically analyzed using SPSS 18.0 software (SPSS Inc., Chicago, IL, United States). Pearson's *χ* ^2^ test was applied to assess the association between Beclin-1 expression and Bcl-2 expression and several clinicopathological variables. The Kaplan–Meier method was used to determine the probability of survival, and the data were analyzed using a log-rank test. The Cox proportional hazards model was used for multivariate analysis of prognostic factors. Comparisons of quantitative data were analyzed using one-way ANOVA for multiple groups. *p*-values <0.05 were considered to be statistically significant.

## Results

### Beclin-1 and Bcl-2 Expression in ESCC

**FIGURE 1:**
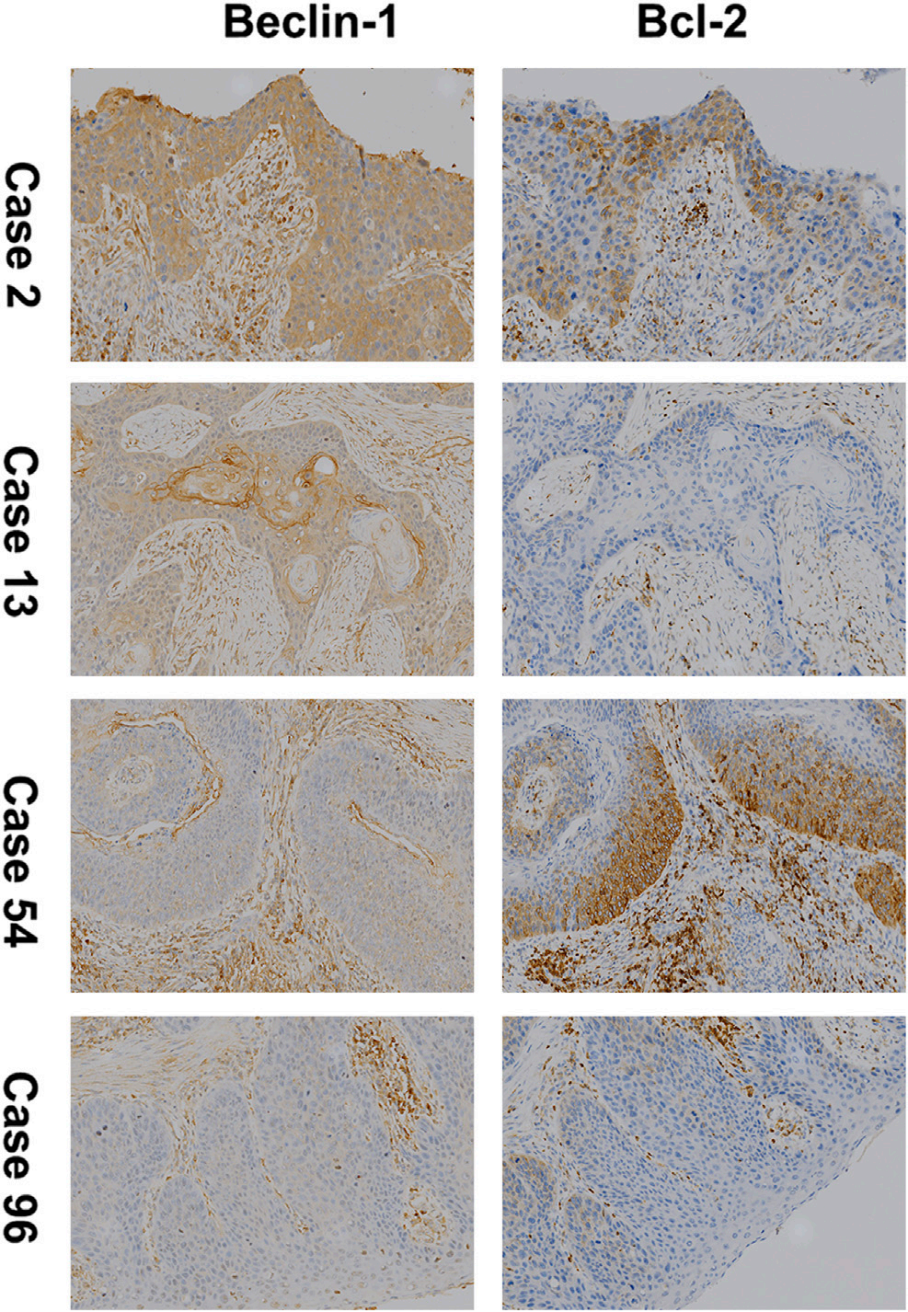
Immunohistochemical staining (100×) for Beclin-1 and Bcl-2 (B-cell lymphoma 2) expression in ESCC. Case 2 for high expression of both; Case 13 for high expression of Beclin-1 and low expression of Bcl-2; Case 54 for low expression of Beclin-1 and high expression of Bcl-2; Case 96 for low expression of both.

The expression of Beclin-1 and Bcl-2 was evaluated in both tumor tissues and adjacent tissues using IHC. As shown in Table 1 34.31% (35/102) of ESCC tissues showed a high expression of Beclin-1. There was also a high expression rate of Beclin-1 in 57.84% (59/102) of adjacent tissues, and this was significantly higher than of the expression in ESCC tissues (*p* < 0.01). In contrast, high expression of Bcl-2 was found in more ESCC tissues than in adjacent tissues (53.92% vs. 32.35% respectively), and this difference was significant. Eventually, we detected that 34.31% (35/102) and 53.92% (55/102) of the ESCC patients showed high expression of Beclin-1 and Bcl-2, respectively. High expression of both Beclin-1 and Bcl-2 was observed in 20 ESCC cases (Figure 1). Furthermore, the expression of Bcl-2 and Beclin-1 in tumor tissues was not correlated (Spearman’s correlation: *p* > 0.05, *r* = 0.047; Table 2).

**TABLE 1:** Expression of Beclin-1 and Bcl-2 in ESCC tissues and adjacent tissues.

Organization type	Beclin-1 expression		*p*	Bcl-2 expression		*p*
	High *n* (%)	Low *n* (%)		High *n* (%)	Low *n* (%)	
ESCC	35 (34.31)	67 (65.69)	<0.01	55 (53.92)	47 (46.08)	<0.01
Adjacent tissues	59 (57.84)	43 (42.16)		33 (32.35)	69 (67.65)	

ESCC: esophageal squamous cell carcinoma; χ^2^ test was applied, *p* < 0.05 was considered to be statistically significant.

**TABLE 2:** Association between Beclin-1 and Bcl-2 expression in ESCC.

Beclin-1	Bcl-2		*p*	*r*
	High expression	Low expression		
High expression	20	15	0.641	0.047
Low expression	35	50

ESCC: esophageal squamous cell carcinoma; *p* < 0.05 was considered to be statistically significant. The relationship between Beclin-1 and Bcl-2 was tested by Pearson’s correlation analysis.

### Correlation of Beclin-1 and Bcl-2 Expression With Clinicopathological Features of ESCC

The relationships between both Beclin-1 and Bcl-2 expression and the clinicopathological features of ESCC patients were analyzed. As shown in Table 3, ESCC with low expression of Beclin-1 was correlated with more advanced stages and lymph node metastasis. In addition, ESCC with high expression of Bcl-2 was correlated with deeper tumor invasion, more advanced stages, and lymph node metastasis. However, the expression of both Beclin-1 and Bcl-2 in ESCC was not associated with age, gender, tumor size, or tumor differentiation.

**TABLE 3:** The relationship between Beclin-1 and Bcl-2 expression with clinicopathological features of ESCC.

Pathologic parameter	Case *n*	Beclin-1 expression		*χ* ^2^	*p*-value	Bcl-2 expression		*χ* ^2^	*p*-value
		High *n* (%)	Low *n* (%)			High *n* (%)	Low *n* (%)		
Gender				0.54	>0.05			0.15	>0.05
Male	65	24 (36.92)	41 (63.08)			36 (55.38)	29 (44.62)		
Female	37	11 (29.73)	26 (70.27)			19 (51.35)	18 (48.65)		
Age				1.07	>0.05			1.07	>0.05
≤60	33	9 (27.27)	24 (72.73)			14 (42.42)	19 (57.58)		
>60	69	26 (37.68)	43 (62.32)			41 (59.42)	28 (40.58)		
Tumour diameter				0.37	>0.05			2.6	>0.05
<3 cm	57	21 (36.84)	36 (63.16)			34 (59.65)	23 (40.35)		
≥3 cm	45	14 (31.11)	31 (68.89)			21 (46.67)	24 (53.33)		
Differentiation				3.5	>0.05			1.83	>0.05
Well and moderate	60	25 (41.67)	35 (58.33)			29 (48.33)	31 (51.67)		
Poor	42	10 (23.81)	32 (76.19)			26 (61.90)	16 (38.10)		
Pathological stage				6.18	<0.05			7.28	<0.01
I–II	44	21 (47.73)	23 (57.27)			17 (38.64)	27 (61.36)		
III–IV	58	14 (24.14)	44 (75.86)			38 (65.52)	20 (34.48)		
Lymph node				7.32	<0.01			7.17	<0.01
Positive	70	18 (25.71)	52 (74.29)			44 (62.86)	26 (37.14)		
Negative	32	17 (53.13)	15 (46.87)			11 (34.38)	21 (65.62)		
Depth of invasion				0.86	>0.05			5.37	<0.05
T1, T2	46	18 (39.13)	28 (60.87)			19 (41.30)	27 (58.70)		
T3, T4	56	17 (30.36)	39 (69.64)			36 (64.29)	20 (35.71)		

*P* < 0.05 was considered statistically significant. χ^2^ test for categorical variables.

### Correlation Between OS and the Changes in Both Beclin-1 and Bcl-2

**FIGURE 2:**
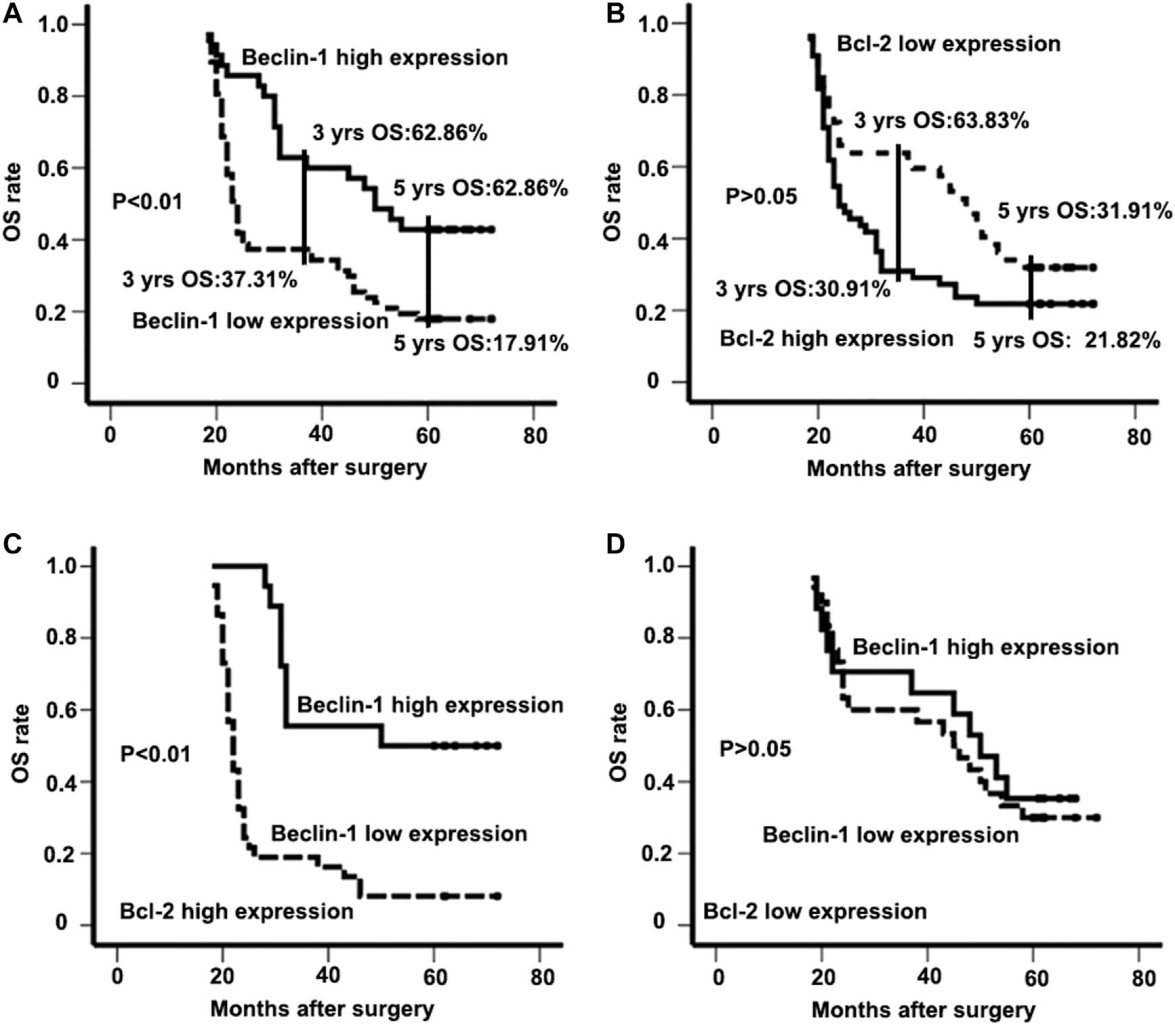
The expression patterns of Beclin-1 and Bcl-2 (B-cell lymphoma 2) in survival analysis of ESCC patients. **(A)** Kaplan-Meier analysis of overall survival for Beclin-1 expression in the whole study population; **(B)** overall survival for Bcl-2 (B-cell lymphoma 2) expression in the whole study population; **(C)** overall survival for the population with high-Bcl-2 (B-cell lymphoma 2) expression; **(D)** overall survival for the population with low- Bcl-2 (B-cell lymphoma 2) expression.

The 3- and 5-years OS of all patients involved was 46.08% and 26.47%, respectively. In patients with high Beclin-1 expression, the 3- and 5-years OS was 62.86% and 42.85%, respectively; in contrast, in patients with low Beclin-1 expression, the 3- and 5-years OS was 37.31% and 17.91%, respectively (Figure 2A). On the other hand, in patients with high Bcl-2 expression, the 3- and 5-years OS was 30.91% and 21.82%, respectively, whereas, in patients with low Bcl-2 expression, the 3- and 5-years OS was 63.83% and 31.91%, respectively (Figure 2B).

### Univariate Analysis and Multivariate Analysis of OS in ESCC Patients

Based on univariate analysis, a more advanced stage (*p* < 0.01), lymph node metastasis (*p* < 0.01), and low Beclin-1 expression (*p* < 0.01) were identified as prognostic factors correlated with poor OS. Further multivariate analysis with these parameters in a Cox regression model confirmed that tumor stage (HR: 18.145; 95% CI: 7.532–43.709; *p* < 0.01), lymph node metastasis (HR: 18.586; 95% CI: 7.021–49.204; *p* < 0.01), and Beclin-1 expression (HR: 2.133; 95% CI: 1.196–3.802; *p* < 0.01; Table 4) were independent prognostic factors associated with OS. We also analyzed the correlation of the combination of Beclin-1 and Bcl-2 expression levels with OS. Cases in which ESCC patients had high expression of both Beclin-1 and Bcl-2 showed significantly longer survival than those with low expression of Beclin-1 (high expression of both Beclin-1 and Bcl-2 group vs. low expression of Beclin-1 and high expression of Bcl-2 group; 52.44 ± 4.72 vs. 27.92 ± 2.44; *p* < 0.01; Figure 2C). When Bcl-2 was in a low-expression state, there was no survival difference in ESCC patients regardless of their expression levels of Beclin-1 (high expression of Beclin-1 and low expression of Bcl-2 group vs. low expression of both Beclin-1 and Bcl-2 group; 46.82 ± 4.75 vs. 44.80 ± 3.86; *p* > 0.05; Figure 2D).

**TABLE 4:** Univariate and multivariate analyses of factors for the prediction of overall survival.

Variable	Univariate analysis HR (95% CI)	*p*-value	Multivariate analysis HR (95% CI)	*p*-value
Gender	1.503 (0.881–2.563)	0.134		
Male				
female				
Age	0.852 (0.483–1.505)	0.582		
≤60				
>60				
Tumor diameter	0.815 (0.491–1.354)	0.430		
<3 cm				
≥3 cm				
Differentiation	1.068 (0.656–1.738)	0.792		
Well and moderate				
Poor				
Pathologic stage	12.944 (4.811–34.826)	<0.01	18.145 (7.532–43.709)	<0.01
I, II				
III, III				
Lymph node	31.281 (10.487–93.305)	<0.01	18.586 (7.021–49.204)	<0.01
Positive				
Negative				
Invade depth	2.048 (0.996–4.213)	0.051		
T1, T2				
T3, T4				
Beclin-1	1.859 (1.020–3.388)	<0.05	2.133 (1.196–3.802)	<0.05
High				
Low				
Bcl-2	1.363 (0.779–2.384)	0.278		
High				
Low				

CI = confidence interval; *p* < 0.05 is considered statistically significant, calculated with continuous variable.

### Differential expression of Beclin-1 and Bcl-2 influences the invasiveness of the EC9706 ESCC cell line

**FIGURE 3:**
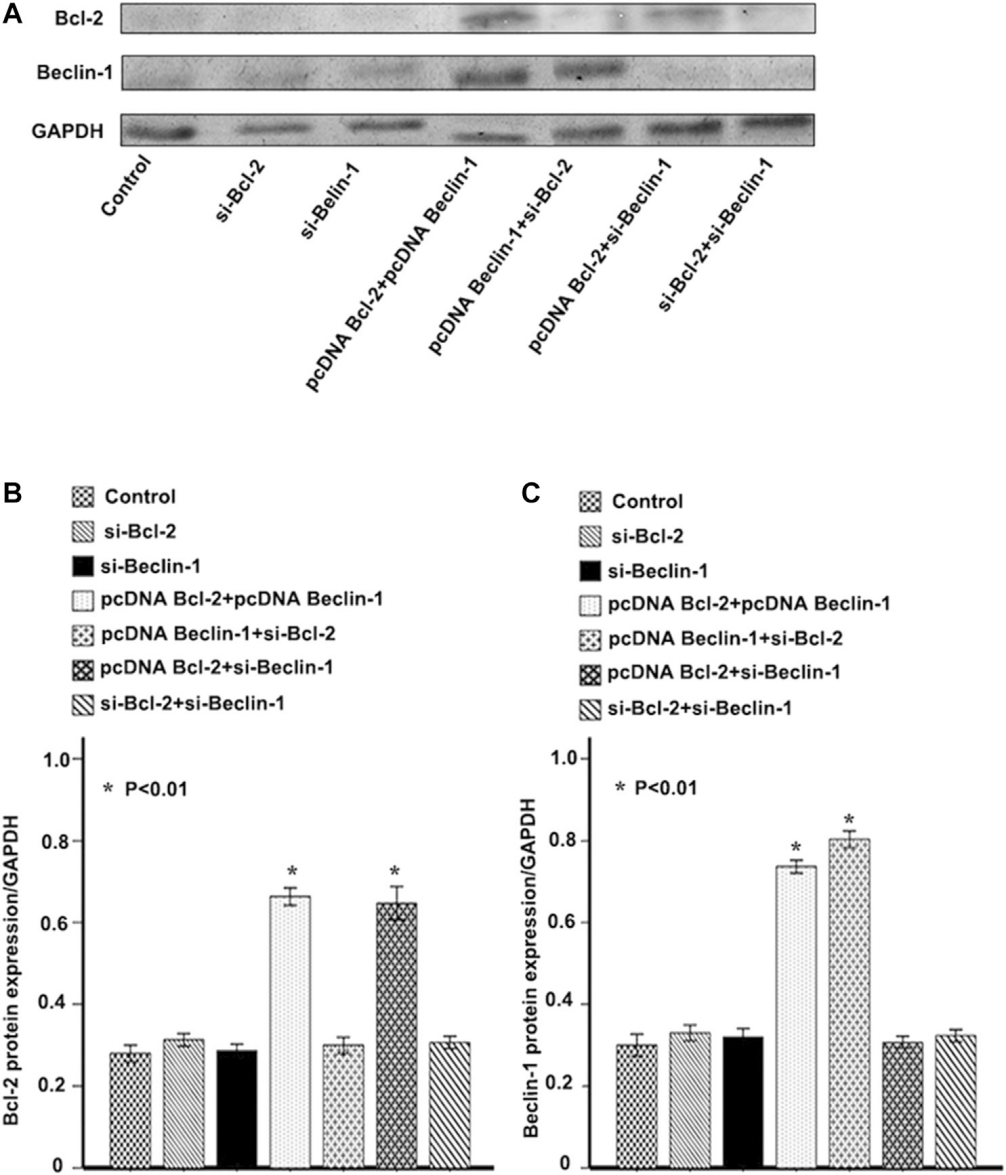
Confirmation of Bcl-2 (B-cell lymphoma 2) and Beclin-1 overexpression or knockdown. **(A)** Western blot analysis of Bcl-2 (B-cell lymphoma 2) and Beclin-1 expression in EC9706 cells transfected with pcDNA3.1-Beclin-1 or Bcl-2 (B-cell lymphoma 2) plasmid and siRNA interference. **(B)** Relative Bcl-2 (B-cell lymphoma 2) protein expression/GAPDH (Glyceraldehyde-3-phosphate dehydrogenase) in each group. **(C)** Relative Beclin-1 protein expression/GAPDH (Glyceraldehyde-3-phosphate dehydrogenase) in each group. *p < 0.01 vs. control group, calculated with one-way ANOVA analysis of variance.

We established EC9706 cells with differential expression levels of Beclin-1 and Bcl-2 by pcDNA3.1-Beclin-1 or Bcl-2 plasmid and siRNA interference. After validation by western blotting (Figure 3), we examined how the different expression level of Beclin-1 and Bcl-2 influence invasiveness. EC9706 cells transfected with pcDNA3.1-Beclin-1 and pcDNA3.1-Bcl-2 showed a significant decrease in the number of invasive cells crossing the polycarbonate membrane of the Transwell invasion chamber (*p* < 0.01). In contrast, EC9706 cells transfected with pcDNA3.1-Bcl-2 and siBeclin-1 showed a significant increase in the number of invasive cells (*p* < 0.01; Table 5). However, the effect of Beclin-1 expression levels on tumor cell invasiveness only appeared in combination with high Bcl-2 expression (there were no observed differences in other groups).

**TABLE 5:** The comparison of invasiveness of EC9706 cells in relation to expression of Beclin-1 and Bcl-2.

Groups	Bcl-2 expression^a^, %	Beclin-1 expression^a^, %	Number of invasive cells
Control	28.00 ± 2.00	30.00 ± 2.65	243.32 ± 38.23
si-Bcl-2	31.33 ± 1.53	33.00 ± 2.00	225.36 ± 28.16
si-Beclin-1	28.67 ± 1.53	32.00 ± 2.00	235.36 ± 31.26
pcDNA Bcl-2 + pcDNA Beclin-1	66.33 ± 2.08	73.67 ± 1.53	52.65 ± 12.24*
pcDNA Beclin-1 + si-Bcl-2	30.00 ± 2.00	80.33 ± 2.08	240.46 ± 32.18
pcDNA Bcl-2 + si-Beclin-1	64.67 ± 4.04	30.67 ± 1.53	381.97 ± 32.31*
si-Bcl-2 + si-Beclin-1	30.67 ± 1.53	32.33 ± 1.53	242.12 ± 36.23

^a^ Mean ± standard deviation, relative to GAPDH; *p < 0.01 vs. untransfected control group, calculated with one-way ANOVA analysis of variance.

## Discussion

As one of the major malignant cancers, esophageal cancer caused about 500,000 deaths worldwide in 2018 [1]. In particular, China has a high incidence of ESCC and a high mortality rate, accounting for about half of all deaths worldwide. Importantly, the incidence of ESCC is increasing, so the disease poses a huge threat to public health and the economy [14–16]. As an important tumor suppressor gene, the study of Beclin-1, which has been identified as a novel Bcl-2-interacting protein, is vital to understanding the role of autophagy in tumorigenesis. In the present study, we investigated the interaction between Bcl-2 and Beclin-1 in ESCC. We found that Beclin-1 expression was associated with tumor stage and lymph node metastasis, and the expression of Beclin-1 in ESCC tissues was significantly lower than that in adjacent tissues, which suggests that autophagy activity was inhibited in ESCC. On the other hand, Bcl-2 expression in ESCC tissues was significantly higher than that in adjacent tissues. These results indicate that Beclin-1 plays an important role in the progression of ESCC and the autophagy defects that occur during more malignant cases of ESCC. Previous research showed that Beclin-1 contains a BH3 domain, which could bind to the BH3 receptor domain of Bcl-2 and further inhibit the Beclin-1-induced autophagy [17]. Our results also suggest that Bcl-2 could bind to Beclin-1 through its receptor domain, which could inhibit autophagy activity and increase the malignancy of ESCC.

Autophagy plays a dual role in protecting and killing tumor cells [18]. Acting as strictly regulated biological processes, autophagy and apoptosis also play an important role in maintaining cell homeostasis [19]. The relationship between autophagy and apoptosis has become a prominent area of research in cancer science. In our study, we explored the important interaction between the autophagy-specific protein Beclin-1 and the anti-apoptotic protein Bcl-2, which are potential targets for autophagy and apoptosis. Therefore, we investigated the combined role of apoptosis and autophagy in the prognosis of ESCC. Immunohistochemical results revealed that the 3- and 5-years OS rates in patients with low Beclin-1 expression were significantly lower than in patients with high Beclin-1 expression. In addition, our analyses indicated that Beclin-1 was an independent predictor of OS in ESCC patients with high Bcl-2 expression. Thus, Beclin-1 should be analyzed in combination with Bcl-2 expression to assess the prognosis of ESCC patients.

In further analysis, we established cells with different Beclin-1 and Bcl-2 expression states using plasmid vectors and siRNA before making *in vitro* observations of the invasiveness of tumor cells. We found that the expression of Beclin-1 significantly affected the number of invasive cells in EC9706 only when Bcl-2 was highly expressed. One of our previous study [20] and another study by Farkas et al. [21] showed that Beclin-1 and Bcl-2 could be prognositc predictive marks in ESCC respectively. Our present study indicate that increased expression of Beclin-1 protein may reduce the invasiveness of EC9706 cells only in combination with high Bcl-2 expression levels. Thus, Beclin-1 must be used as an independent prognostic factor for ESCC patients in combination with Bcl-2 expression. On the other hand, our study also raises the following question: why were different Beclin-1 expression levels not relative to invasiveness and prognosis in ESCC patients with low Bcl-2 expression? Previous research has shown that apoptosis could eliminate tumor cells [22, 23]. In addition, autophagy is known to play a role in protecting tumor cell survival in tumorigenesis and progression with intact apoptosis [24, 25]. Furthermore, autophagic cell death may only occur when apoptosis is inhibited [23, 26]. Our results suggest that a relationship between autophagy and apoptosis may also exist in ESCC. Of course, we cannot eliminate other complex molecular mechanisms and signaling pathways as potential regulators in the occurrence and progression of ESCC. However, the present study’s limitation is that it is a retrospective analysis of a comparatively small sample, other studies and potential mechanisms must therefore be evaluated in future research studies.

In summary, we have shown that autophagy-specific protein expression and autophagy activity were inhibited in ESCC. Differences in autophagy and apoptotic states and their activities may promote malignant tumor differentiation, which in turn may lead to a more aggressive esophageal squamous cell phenotype and a worse survival prognosis. Importantly, Beclin-1 is a promising prognostic biomarker and therapeutic target for patients with ESCC in the high-Bcl-2-expression population.

## Data Availability

The original contributions presented in the study are included in the article/Supplementary Material, further inquiries can be directed to the corresponding author.
